# Anemia and Red-Cell Size Across Estimated Glomerular Filtration Rate in a Hospital Laboratory Cohort

**DOI:** 10.3390/jcm15208120

**Published:** 2026-10-19

**Authors:** Yahya Madkhali

**Affiliations:** Department of Medical Laboratory Sciences, College of Applied Medical Sciences, Majmaah University, Al Majmaah City 11952, Saudi Arabia; y.madkhali@mu.edu.sa

**Keywords:** anemia, estimated glomerular filtration rate, mean corpuscular volume, kidney function, routine laboratory data

## Abstract

**Background/Objective**: Anemia patterns may differ between population and hospital-tested cohorts. This paper aims to examine anemia frequency and red-cell size across estimated glomerular filtration rate (eGFR) in hospital records. **Methods**: This retrospective cross-sectional study included 37,831 adults aged 18–100 years tested at King Khalid Hospital, Saudi Arabia, during 2017–2021. For each individual, one complete blood count (CBC) record and one creatinine record from the same calendar month were analyzed. eGFR was calculated using the 2021 CKD-EPI creatinine equation. Modified Poisson, logistic and natural cubic spline models adjusted for age, sex, and calendar year. **Results**: Anemia occurred in 8762 participants (23.2%): 22.9%, 17.1%, 34.6%, and 72.1% at eGFR ≥ 90, 60–89, 30–59, and <30 mL/min/1.73 m^2^, respectively. Relative to eGFR 60–89, adjusted prevalence ratios (PRs; 95% confidence intervals [CIs]) were 1.26 (1.19–1.33) for ≥90, 1.90 (1.75–2.07) for 30–59, and 3.98 (3.69–4.30) for <30. The association was nonlinear (*p* < 0.001). Among participants with anemia, the normocytic proportion increased from 56.6% at eGFR ≥ 90 to 82.7% at <30. In men, the adjusted normocytic-versus-microcytic odds ratio (OR) for <30 versus 60–89 was 2.70 (1.71–4.27). **Conclusions**: eGFR < 60 was associated with more frequent anemia. The proportion of anemia classified as normocytic was also greater in lower eGFR categories. Based on same-month CBC and creatinine records, these associations do not confirm CKD or determine anemia etiology.

## 1. Introduction

Anemia is a common complication of chronic kidney disease (CKD) [1]. Population studies have found that anemia becomes more frequent as kidney function declines [2,3]. More recent US population and outpatient cohorts have also reported more frequent anemia at lower estimated glomerular filtration rate (eGFR) [4,5]. The magnitude of the association varies with the population studied, the definition of anemia, and how kidney disease is identified [2,3].

CKD-associated anemia is often normocytic and hypoproliferative [6]. Reduced erythropoietin production relative to the degree of anemia and impaired iron availability are established contributors [6]. Inflammation can restrict iron delivery to the marrow through increased hepcidin activity [7]. Shortened red-cell survival may also contribute. In a study of 122 patients with nondialysis CKD, lower eGFR was associated with greater annexin V binding, a marker of red-cell death [8]. These mechanisms cannot be distinguished from routine hemoglobin and mean corpuscular volume (MCV) measurements alone.

The 2021 Chronic Kidney Disease Epidemiology Collaboration (CKD-EPI) creatinine equation estimates GFR without a race coefficient [9]. Creatinine also depends on factors other than filtration, including muscle mass, and its interpretation is less reliable when kidney function is changing rapidly [10,11]. A single abnormal eGFR does not establish CKD; evidence of chronicity is required [10]. These limitations matter when interpreting anemia patterns in hospital laboratory records.

International CKD cohorts have described differences in hemoglobin distributions across regions [12]. Saudi research has also described hematologic reference intervals in screened adults, but those data address a different question from anemia among people undergoing hospital testing [13]. The present study examined anemia frequency and MCV-defined red-cell size across eGFR categories in a Saudi hospital laboratory dataset. It also assessed the continuous eGFR–anemia relationship and the stability of the associations under available linkage restrictions and age and sex subgroup analyses.

## 2. Materials and Methods

### 2.1. Study Design and Data Source

This retrospective cross-sectional study used laboratory records from King Khalid Hospital, Al-Majmaah, Saudi Arabia (1 January 2017–31 December 2021). The dataset included 37,831 adults aged 18–100 years with sex, age, hemoglobin, MCV, and serum creatinine. All eligible individuals were included. No prospective sample-size calculation was performed. Eligible ranges were hemoglobin 2–25 g/dL, MCV 40–130 fL, and creatinine 20–2000 µmol/L. Patient setting (inpatient, outpatient or emergency), dialysis or transplant status, and erythropoiesis-stimulating agent or iron treatment were not available in the analysis extract. Creatinine measurements beyond the index month were unavailable to assess chronicity.

### 2.2. Record Linkage and Index Selection

CBC and biochemistry records were linked by study identifier, calendar year, and calendar month from the source file name. For each individual, the earliest eligible period was selected by year and numeric file order. File label and source-row order resolved ties. The first eligible creatinine result in source-row order was the index measurement. Each index month had one eligible CBC record; 36,188 participants also had exactly one biochemistry record and formed the restricted-linkage sensitivity sample. Selections and counts were checked against the source workbook. Linked tests were from the same month; without exact dates, same-day testing could not be confirmed. Linkage within 48–72 h was therefore not possible.

### 2.3. Laboratory Measurements and Definitions

Biochemical measurements were performed using a Beckman Coulter DxC 700 AU analyzer (Beckman Coulter, Inc., Brea, CA, USA), and hematological measurements were performed using an Abbott CELL-DYN Ruby analyzer (Abbott Laboratories, Abbott Park, IL, USA). Serum creatinine was measured using an enzymatic assay with calibration traceable to isotope-dilution mass spectrometry (IDMS) and a creatinine reference interval of 53–120 µmol/L for both sexes. Creatinine was converted from µmol/L to mg/dL by dividing by 88.4. eGFR was calculated from creatinine, age, and recorded sex with the 2021 CKD-EPI creatinine equation without a race coefficient [9]. eGFR categories were ≥90, 60–89, 30–59, and <30 mL/min/1.73 m^2^. These were eGFR categories, not CKD stages, because chronicity and other evidence of kidney damage were unavailable [10]. Anemia was defined as hemoglobin <12 g/dL in women and <13 g/dL in men, based on the 2024 World Health Organization thresholds for nonpregnant women and men aged 15–65 years [14]. The WHO guideline (normative statement 1.a) does not specify cutoffs above age 65. The adult cutoffs were extended above 65, with a sensitivity analysis restricted to ages 18–65. Data on pregnancy, smoking, and altitude were unavailable. Among participants with anemia, MCV categories were microcytic (<80 fL), normocytic (80–100 fL), and macrocytic (>100 fL); these describe red-cell size, not the cause of anemia.

### 2.4. Primary and Continuous Analyses

Continuous variables are reported as median (IQR), categorical variables as number (percentage), and observed anemia proportions with Wilson 95% confidence intervals (CIs). Anemia proportions were directly standardized to the total cohort’s joint age–sex distribution, using age bands of 18–39, 40–49, 50–59, 60–69, and ≥70 years. Standardized CIs used a delta-method logit transformation with fixed reference weights. Modified Poisson regression with robust standard errors provided adjusted prevalence ratios, adjusting for sex, age (per 10 years), and calendar year. Logistic odds ratios (ORs) were secondary [15]. Reference groups were eGFR 60–89 mL/min/1.73 m^2^, male sex, and 2017. Results are adjusted prevalence ratios (PRs) with 95% CIs.

Continuous eGFR was modeled by logistic regression with a four-degree-of-freedom natural cubic spline, using interior knots at the 25th, 50th, and 75th percentiles and boundary knots at the observed limits. A three-degree-of-freedom likelihood-ratio test compared spline and linear models adjusted for sex, age, and year. Predicted anemia probabilities were averaged over the observed covariate distribution; pointwise 95% CIs were calculated by the delta method on the logit scale. Models used the full eGFR range; the displayed curve covered the 1st–99th eGFR percentiles, with the full-range curve in Figure S1, and its minimum was not treated as a clinical threshold. Rug marks summarize counts in 0.5-unit eGFR bins, with darker marks indicating more observations. Among participants with anemia, a logistic model compared normocytic with microcytic anemia across four eGFR categories, with 60–89 mL/min/1.73 m^2^ as the reference. It adjusted for sex, age, and year; 128 participants with macrocytic anemia were excluded. Morphology models were also fitted separately in men and women, adjusting for age and year, and at ages <50 and ≥50, adjusting for sex, age and year.

### 2.5. Classification of Anemia Severity

Among participants with anemia, severity was classified as mild (hemoglobin 11 to <12 g/dL in women or 11 to <13 g/dL in men), moderate (8 to <11 g/dL), or severe (<8 g/dL), following normative statement 1.b and Table 3 of the WHO guideline [14]. These adult thresholds were extended above age 65, with severity summarized separately for ages 18–65 and >65.

### 2.6. Sensitivity Analyses and Software

Sensitivity analyses restricted records to months with one eligible CBC and one biochemistry record, excluded eGFR > 130 mL/min/1.73 m^2^, or limited age to 18–65 years. Other models modeled age with a natural cubic spline using knots at its 25th, 50th, and 75th percentiles. Sex-specific models adjusted for age and year. The eGFR-by-sex interaction was assessed using a three-degree-of-freedom robust Wald test with sex-specific age and year effects. Secondary analyses were exploratory, without adjustment for multiple comparisons. Wald tests assessed individual coefficients. Likelihood-ratio tests compared nested models. All tests were two-sided. *p* < 0.05 was the reporting threshold. Analyses used R 4.4.1 (stats and splines). Reporting was checked against STROBE and RECORD guidance [16,17].

### 2.7. Ethics

The King Fahad Medical City Institutional Review Board classified this study as exempt on 2 November 2022 (IRB Log No. 22-485E). Direct identifiers were excluded, and the study followed the Declaration of Helsinki [18].

## 3. Results

### 3.1. Analytic Population

The cohort included 37,831 adults, of whom 20,161 (53.3%) were women. Median age was 40 years (IQR, 29–55), and median eGFR was 106.3 mL/min/1.73 m^2^ (IQR, 88.3–120.6). All core analysis variables were complete in the retained extract. Anemia was present in 8762 participants (23.2%). Characteristics across eGFR categories are shown in Table 1.

The stored biochemistry count was one for 36,188 index periods (95.7%) and two to four for the remaining 1643 periods.

### 3.2. Anemia Across eGFR Categories

**Figure 1:**
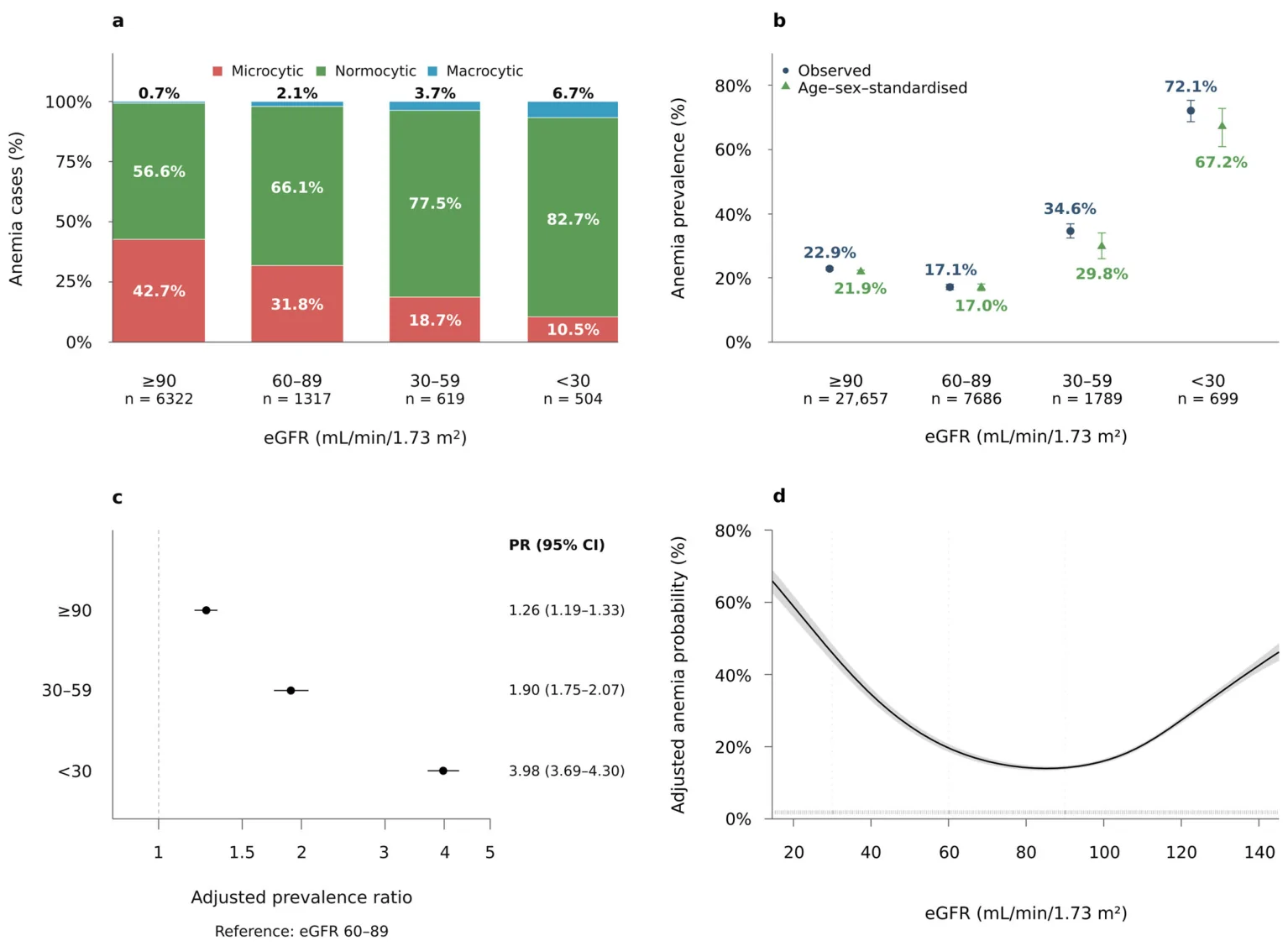
Anemia frequency, red-cell size and adjusted associations across eGFR. (**a**) Distribution of microcytic, normocytic and macrocytic categories among participants with anemia across eGFR categories; *n* denotes anemia cases. (**b**) Observed and age–sex-standardized anemia proportions with 95% confidence intervals; *n* denotes all participants in each category. Observed intervals are Wilson intervals; standardized intervals use a delta-method logit transformation with fixed full-cohort reference weights. (**c**) Adjusted prevalence ratios with robust 95% confidence intervals from the primary model, relative to eGFR 60–89, on a logarithmic axis. The vertical dashed line indicates PR = 1, representing no difference in anemia prevalence relative to the reference category. (**d**) Adjusted predicted probability of anemia across continuous eGFR values, averaged over the observed covariate distribution; gray shading shows pointwise 95% confidence intervals. The model used all participants; the main display spans the 1st–99th eGFR percentiles. The full-range curve is in Figure S1. Rug marks represent counts in 0.5-unit eGFR bins, with darker marks indicating more observations. Dotted lines mark eGFR 30, 60 and 90 mL/min/1.73 m^2^. eGFR, estimated glomerular filtration rate.

Anemia was present in 6322 of 27,657 participants with eGFR ≥ 90 (22.9%), 1317 of 7686 with eGFR 60–89 (17.1%), 619 of 1789 with eGFR 30–59 (34.6%), and 504 of 699 with eGFR < 30 mL/min/1.73 m^2^ (72.1%) (Table 1; Figure 1b). Age–sex-standardized prevalence was 21.9%, 17.0%, 29.8%, and 67.2%, respectively.

### 3.3. Red-Cell Size Among Participants with Anemia

Across all participants in each category, median hemoglobin was 13.5, 14.2, 13.3, and 11.1 g/dL at eGFR ≥ 90, 60–89, 30–59, and <30, respectively. Among participants with anemia, the normocytic proportion increased from 56.6% at eGFR ≥ 90 to 82.7% at eGFR < 30, while the microcytic proportion decreased from 42.7% to 10.5% (Table 2; Figure 1a). At eGFR < 30, 417 of 504 participants with anemia were normocytic, 53 were microcytic, and 34 were macrocytic.

The normocytic-versus-microcytic model included 8634 participants. Relative to eGFR 60–89, adjusted ORs were 0.90 (95% CI, 0.78–1.04; *p* = 0.140) for eGFR ≥ 90, 1.75 (1.37–2.22; *p* < 0.001) for 30–59, and 3.49 (2.56–4.77; *p* < 0.001) for <30 (Table S2). In men, the normocytic proportion increased from 64.3% at eGFR ≥ 90 to 83.3% at <30; the adjusted OR for <30 versus 60–89 was 2.70 (95% CI, 1.71–4.27). The corresponding ORs were 4.32 (2.81–6.65) in women, 3.58 (2.02–6.36) at age < 50, and 3.36 (2.30–4.89) at age ≥ 50. Distributions by sex and age are shown in Table 2, with subgroup model estimates in Table S2.

### 3.4. Adjusted eGFR Association

After adjustment for age, sex, and calendar year, the PR was 1.26 (95% CI, 1.19–1.33) for eGFR ≥ 90, 1.90 (1.75–2.07) for eGFR 30–59, and 3.98 (3.69–4.30) for eGFR < 30 relative to 60–89 (all *p* < 0.001). The PR for women versus men was 2.56 (95% CI, 2.45–2.67), and the PR per 10-year increase in age was 1.04 (1.02–1.05; *p* < 0.001) (Table 3). Secondary ORs are shown in Table S2. Relative to 2017, adjusted PRs were 1.09, 1.43, 1.15, and 1.29 for 2018–2021, respectively; this calendar-year pattern was not monotonic.

### 3.5. Continuous eGFR Association

The continuous association was nonlinear (likelihood-ratio statistic, 1886.31; three degrees of freedom; *p* < 0.001). The lowest displayed adjusted probability was 14.0% near eGFR 85 mL/min/1.73 m^2^. Predicted probabilities were 46.0% at eGFR 30 and 19.6% at eGFR 60. The curve also rose at high eGFR (Figure 1d). There were 3582 participants (9.5%) with eGFR > 130; their median age was 23 years, median creatinine was 46 µmol/L, and observed anemia proportion was 32.8%. Creatinine was below the laboratory’s lower reference limit of 53 µmol/L in 7318 of 37,831 participants (19.3%), including 2536 of the 3582 participants with eGFR > 130 (70.8%). The nonlinear association persisted with flexible age adjustment (*p* < 0.001).

### 3.6. Anemia Severity

Among the 8762 participants with anemia, 4766 (54.4%) had mild, 3594 (41.0%) moderate, and 402 (4.6%) severe anemia. At eGFR < 30, 274 of 504 anemia cases (54.4%) were moderate and 59 (11.7%) were severe (Table 4). Severity distributions are shown separately for ages 18–65 and >65 in Table 4.

### 3.7. Sensitivity Analyses

Restriction to 36,188 index records with one eligible CBC and one eligible biochemistry record gave adjusted PRs of 1.95 (95% CI, 1.79–2.12) for eGFR 30–59 and 4.12 (3.80–4.47) for <30, relative to 60–89. Excluding eGFR > 130 retained 34,249 participants and gave PRs of 1.84 (1.69–2.00) and 3.88 (3.60–4.20). In the 33,142 participants aged 18–65, the corresponding PRs were 1.96 (1.72–2.22) and 4.87 (4.36–5.43). Flexible age adjustment gave PRs of 1.85 (1.70–2.02) and 3.92 (3.62–4.24) (Table S3).

In separately fitted models, the PR for eGFR < 30 versus 60–89 was 4.73 (95% CI, 4.24–5.28) in men and 3.35 (3.05–3.68) in women. The eGFR-by-sex robust Wald test, allowing age and year associations to differ by sex, gave *p* < 0.001 (Table S4).

## 4. Discussion

In this hospital laboratory dataset, anemia was much more frequent among participants with eGFR < 60 mL/min/1.73 m^2^ and affected 72.1% of those with eGFR < 30. The proportion of anemia classified as normocytic was also greater in lower eGFR categories. The association remained within men and both age groups, although demographic and clinical differences may still contribute. The main low-eGFR associations remained similar after restriction to index records with one eligible CBC and one eligible biochemistry record, alternative age adjustment, and exclusion of high eGFR values.

The direction of the association agrees with the National Health and Nutrition Examination Survey analysis by Stauffer and Fan and the Danish study by Vestergaard and colleagues [2,3]. Their designs differ from the present analysis: the former used a population survey, while the latter followed patients with newly identified CKD. The International Network of CKD Cohorts analysis also found regional variation in hemoglobin distributions among patients with CKD [12]. These differences in population, methods used to identify kidney disease and treatment make direct comparisons of anemia frequency difficult. The present study describes this association in linked CBC and creatinine records from a Saudi hospital.

The normocytic pattern is compatible with several processes described in CKD, including an inadequate erythropoietin response and inflammation-related iron restriction, but it does not confirm either mechanism [6,7]. Acute illness can produce a similar pattern, and MCV alone cannot identify renal anemia. A falling proportion of microcytosis also does not exclude iron deficiency, particularly when causes coexist [7]. Alpha-thalassemia can be associated with microcytosis despite normal ferritin [19]. Beta-thalassemia and sickle cell disorders are also documented in Saudi national premarital screening data [20]. Macrocytosis has multiple possible causes, none of which could be established here [21].

Serum iron alone does not provide a complete assessment of iron status [1]. Incomplete iron-status testing has also been reported in multinational nephrology cohorts [22]. A large US CKD cohort classified absolute and functional iron deficiency using both ferritin and transferrin saturation [23]. Those measurements were unavailable here, so serum iron could not distinguish these categories.

Anemia frequency was lower at eGFR 60–89 than at ≥90, and the adjusted spline had a minimum near 85 mL/min/1.73 m^2^. Neither observation establishes an optimal eGFR or a protective effect of mildly lower filtration. Creatinine-based eGFR can be influenced by muscle mass and other nonfiltration factors [10,11]. In a longitudinal cohort, low muscle mass was associated with overestimation of creatinine-based eGFR [24]. Muscle mass was not measured in the present study. The high-eGFR portion of the curve could also reflect differences in illness, testing indications, or population composition. Diabetes-related hyperfiltration and reduced creatinine production from severe wasting or malnutrition are possible explanations that could not be confirmed or distinguished using creatinine-based eGFR alone [24,25]. Its shape and minimum depend on the model specification. The analysis excluding eGFR > 130 supports the stability of the low-eGFR associations but does not validate the high-eGFR curve.

Women had a higher overall prevalence of meeting the sex-specific anemia definition, whereas the relative low-eGFR association was larger in the separately fitted model for men. Because anemia was defined using different hemoglobin cutoffs for women and men, the female-to-male PR does not compare the sexes at a common threshold. The interaction was measured on the prevalence-ratio scale and does not establish a stronger biological effect in men. Pregnancy, menstrual history, treatment, and complete iron-status measurements were unavailable. Nonmonotonic calendar-year associations may reflect differences in the tested population or clinical setting, but their cause could not be established.

### Strengths and Limitations

The sample size gave adequate precision for estimating the main associations and comparing MCV-defined red-cell size categories. No data were missing for the core variables. eGFR and anemia status were recalculated, with a single record retained for each coded participant. For the sensitivity analysis, the models were rerun after removing index periods containing more than one biochemistry record.

Several limitations remain. Referral and testing practices at a single hospital may limit generalizability, so the reported anemia frequencies are not population prevalence estimates. Patient setting was unavailable, and acute illness, bleeding, hemodilution or acute kidney injury may affect hemoglobin and creatinine, limiting comparison with stable outpatient cohorts. CBC and creatinine results were linked within the same month, but exact testing intervals were unknown. Changes between tests may cause misclassification, and the single-record restriction does not establish temporal agreement. A single creatinine-based eGFR cannot confirm CKD or distinguish chronic from acute kidney dysfunction [10]. Dialysis and transplant patients could not be identified or excluded because status was unavailable, and erythropoiesis-stimulating agent and iron-treatment data were also unavailable.

Incomplete clinical, treatment and laboratory data limited assessment of other causes of anemia and potential confounding, with RDW, hemoglobin electrophoresis, HbA2 and premarital screening results unavailable in the extract. The use of the same sex-specific cutoffs across adult ages may have affected classification in older adults, although the age-restricted analysis gave similar results. Pregnancy status was unavailable and may also have affected classification. MCV comparisons included only participants with anemia and cannot determine its cause. The cross-sectional design cannot establish temporal order or causality.

## 5. Conclusions

Anemia was more frequent among participants with eGFR < 60 mL/min/1.73 m^2^, and among those with anemia, the proportion with an MCV-defined normocytic pattern was greater in lower eGFR categories. Low-eGFR associations remained similar across sensitivity analyses, and the normocytic-versus-microcytic association remained within men and both age groups. These cross-sectional laboratory associations do not establish CKD, renal anemia, or a clinical eGFR threshold.

## Figures and Tables

**Table 1:** Characteristics across eGFR categories.

Characteristic	≥90	60–89	30–59	<30
Participants, *n*	27,657	7686	1789	699
Age, years	36 (27–47)	54 (40–66)	67 (56–78)	63 (52–77)
Men, *n* (%)	11,937 (43.2)	4399 (57.2)	964 (53.9)	370 (52.9)
Women, *n* (%)	15,720 (56.8)	3287 (42.8)	825 (46.1)	329 (47.1)
Creatinine, µmol/L	62.0 (52.0–74.0)	91.0 (79.0–102.0)	124.0 (108.0–142.0)	371.0 (235.5–649.0)
Hemoglobin, g/dL	13.5 (12.2–15.0)	14.2 (12.8–15.4)	13.3 (11.8–14.6)	11.1 (9.5–12.7)
MCV, fL	86.5 (82.3–90.1)	87.6 (83.8–91.1)	87.9 (83.8–91.9)	89.3 (85.4–94.2)
Anemia, *n* (%)	6322 (22.9)	1317 (17.1)	619 (34.6)	504 (72.1)
Age–sex-standardized anemia, % (95% CI)	21.9 (21.4–22.4)	17.0 (16.0–18.1)	29.8 (26.0–34.0)	67.2 (60.9–72.8)

eGFR categories are in mL/min/1.73 m^2^. Values are median (IQR) or number (%). Percentages use all participants within each category. Standardized prevalence uses the full cohort’s joint sex and age distribution; intervals use fixed reference weights. eGFR, estimated glomerular filtration rate; IQR, interquartile range; MCV, mean corpuscular volume.

**Table 2:** Red-cell size among participants with anemia, overall and by sex and age.

eGFR Category	Anemia, *n*	Microcytic, *n* (%)	Normocytic, *n* (%)	Macrocytic, *n* (%)
**Overall**				
≥90	6322	2699 (42.7)	3579 (56.6)	44 (0.7)
60–89	1317	419 (31.8)	871 (66.1)	27 (2.1)
30–59	619	116 (18.7)	480 (77.5)	23 (3.7)
<30	504	53 (10.5)	417 (82.7)	34 (6.7)
**Men**				
≥90	1128	381 (33.8)	725 (64.3)	22 (2.0)
60–89	550	124 (22.5)	412 (74.9)	14 (2.5)
30–59	299	42 (14.0)	245 (81.9)	12 (4.0)
<30	263	26 (9.9)	219 (83.3)	18 (6.8)
**Women**				
≥90	5194	2318 (44.6)	2854 (54.9)	22 (0.4)
60–89	767	295 (38.5)	459 (59.8)	13 (1.7)
30–59	320	74 (23.1)	235 (73.4)	11 (3.4)
<30	241	27 (11.2)	198 (82.2)	16 (6.6)
**Age < 50 years**				
≥90	5116	2251 (44.0)	2842 (55.6)	23 (0.4)
60–89	440	195 (44.3)	242 (55.0)	3 (0.7)
30–59	73	22 (30.1)	49 (67.1)	2 (2.7)
<30	100	16 (16.0)	81 (81.0)	3 (3.0)
**Age ≥ 50 years**				
≥90	1206	448 (37.1)	737 (61.1)	21 (1.7)
60–89	877	224 (25.5)	629 (71.7)	24 (2.7)
30–59	546	94 (17.2)	431 (78.9)	21 (3.8)
<30	404	37 (9.2)	336 (83.2)	31 (7.7)

eGFR, estimated glomerular filtration rate; MCV, mean corpuscular volume. eGFR categories are in mL/min/1.73 m^2^. Microcytic: MCV < 80 fL; normocytic: MCV 80–100 fL; macrocytic: MCV > 100 fL. Percentages use participants with anemia in each subgroup and eGFR category and may not sum to 100 because of rounding. Sex and age panels overlap.

**Table 3:** Adjusted associations with laboratory-defined anemia.

Variable	Adjusted PR	95% CI	*p* Value
Female vs. male	2.56	2.45–2.67	<0.001
Age per 10 years	1.04	1.02–1.05	<0.001
eGFR ≥90 vs. 60–89	1.26	1.19–1.33	<0.001
eGFR 30–59 vs. 60–89	1.90	1.75–2.07	<0.001
eGFR <30 vs. 60–89	3.98	3.69–4.30	<0.001
2018 vs. 2017	1.09	1.03–1.15	0.003
2019 vs. 2017	1.43	1.35–1.51	<0.001
2020 vs. 2017	1.15	1.08–1.22	<0.001
2021 vs. 2017	1.29	1.22–1.37	<0.001

N = 37,831. The model included all listed terms. Reference categories were male sex, eGFR 60–89 mL/min/1.73 m^2^ and 2017. Age was modeled per 10-year increase. Modified Poisson models used HC0 robust 95% CIs. eGFR, estimated glomerular filtration rate; PR, prevalence ratio; CI, confidence interval.

**Table 4:** Anemia severity across eGFR categories.

eGFR Category	Participants, *n*	Anemia, *n*	Mild, *n* (%)	Moderate, *n* (%)	Severe, *n* (%)
**Overall**					
≥90	27,657	6322	3436 (54.3)	2622 (41.5)	264 (4.2)
60–89	7686	1317	811 (61.6)	458 (34.8)	48 (3.6)
30–59	1789	619	348 (56.2)	240 (38.8)	31 (5.0)
<30	699	504	171 (33.9)	274 (54.4)	59 (11.7)
**Ages 18–65 years**					
≥90	26,282	6055	3255 (53.8)	2543 (42.0)	257 (4.2)
60–89	5673	818	496 (60.6)	294 (35.9)	28 (3.4)
30–59	805	231	128 (55.4)	93 (40.3)	10 (4.3)
<30	382	265	100 (37.7)	126 (47.5)	39 (14.7)
**Age > 65 years**					
≥90	1375	267	181 (67.8)	79 (29.6)	7 (2.6)
60–89	2013	499	315 (63.1)	164 (32.9)	20 (4.0)
30–59	984	388	220 (56.7)	147 (37.9)	21 (5.4)
<30	317	239	71 (29.7)	148 (61.9)	20 (8.4)

eGFR categories are in mL/min/1.73 m^2^. Percentages use participants with anemia in each age group and eGFR category. Mild: hemoglobin 11 to <12 g/dL in women or 11 to <13 g/dL in men; moderate: 8 to <11 g/dL; severe: <8 g/dL. WHO adult thresholds were extended analytically above age 65. Pregnancy status was unavailable. eGFR, estimated glomerular filtration rate.

## Data Availability

The analyzed summary data are presented within the article and Supplementary Materials. Record-level data are not publicly available because they contain sensitive clinical information and are subject to institutional and ethical restrictions.

## Supplementary Materials

The following supporting information can be downloaded at https://www.mdpi.com/article/10.3390/jcm15208120/s1, Table S1. Biomarker cleaning counts. Table S2. Four-category anemia and red-cell-size models. Table S3. Sensitivity analyses. Table S4. Sex and age subgroup models. Table S5. Composition of complete-case biomarker subsets. Figure S1. Full-range association between eGFR and anemia.
