# Supplementary material for: Anemia and Red-Cell Size Across Estimated Glomerular Filtration Rate in a Hospital Laboratory Cohort
Source: J Clin Med. 2026 Oct 19;15(20):8120. doi: 10.3390/jcm15208120 (PMC13706170; doi:10.3390/jcm15208120)
Supplement: Supplementary file 1 [file jcm-15-08120-s001.zip › jcm-4622317-supplementary.pdf]

Supplementary material

Supplementary Methods

For the continuous eGFR model, interior knots were 88.264, 106.279, and 120.575 mL/min/1.73 m²; boundary knots were 1.627 and 192.650 mL/min/1.73 m². Table S1 documents the prespecified numerical cleaning limits and available biomarker counts. Complete-case subsets were formed after values outside these limits were set to missing. Tables S2–S5 report the additional model estimates and subset composition. Morphology was also compared within men, women, ages <50 and ages ≥50. Sex-specific models adjusted for age and year; age-specific models adjusted for sex, age and year. All used eGFR 60–89 as the reference and excluded macrocytic anemia. The full-range spline display is provided in Figure S1.

Table S1. Biomarker cleaning counts

Biomarker	Lower	Upper	Unit	Available before cleaning	Outside bounds	Retained
Urea	0.5	100	mmol/L	34,553	4	34,549
Albumin	5	70	g/L	11,812	17	11,795
Iron	2	500	µg/dL	8,366	40	8,326
Calcium	0.5	4	mmol/L	11,710	26	11,684
Phosphorus	0.1	6	mmol/L	8,564	7	8,557
HbA1c	3	25	%	4,568	22	4,546

Bounds are inclusive and use the units stated in the supplied dictionary. Values outside these bounds were set to missing; no biomarker unit conversions were applied. HbA1c, glycated hemoglobin.

Table S2. Four-category anemia and red-cell-size models

Model	N	Comparison	Adjusted OR (95% CI)	P value
Anemia	37,831	eGFR ≥90 vs 60–89	1.35 (1.26–1.46)	<0.001
Anemia	37,831	eGFR 30–59 vs 60–89	2.52 (2.24–2.84)	<0.001
Anemia	37,831	eGFR <30 vs 60–89	14.70 (12.23–17.67)	<0.001
Normocytic vs microcytic	8,634	eGFR ≥90 vs 60–89	0.90 (0.78–1.04)	0.140
Normocytic vs microcytic	8,634	eGFR 30–59 vs 60–89	1.75 (1.37–2.22)	<0.001
Normocytic vs microcytic	8,634	eGFR <30 vs 60–89	3.49 (2.56–4.77)	<0.001
Men (morphology)	2,174	eGFR ≥90 vs 60–89	0.80 (0.61–1.06)	0.115
Men (morphology)	2,174	eGFR 30–59 vs 60–89	1.60 (1.08–2.36)	0.018
Men (morphology)	2,174	eGFR <30 vs 60–89	2.70 (1.71–4.27)	<0.001
Women (morphology)	6,460	eGFR ≥90 vs 60–89	0.95 (0.80–1.12)	0.532
Women (morphology)	6,460	eGFR 30–59 vs 60–89	1.84 (1.36–2.50)	<0.001
Women (morphology)	6,460	eGFR <30 vs 60–89	4.32 (2.81–6.65)	<0.001
Age <50 (morphology)	5,698	eGFR ≥90 vs 60–89	1.06 (0.86–1.30)	0.580
Age <50 (morphology)	5,698	eGFR 30–59 vs 60–89	1.78 (1.04–3.05)	0.037
Age <50 (morphology)	5,698	eGFR <30 vs 60–89	3.58 (2.02–6.36)	<0.001
Age ≥50 (morphology)	2,936	eGFR ≥90 vs 60–89	0.82 (0.66–1.01)	0.064
Age ≥50 (morphology)	2,936	eGFR 30–59 vs 60–89	1.54 (1.17–2.03)	0.002
Age ≥50 (morphology)	2,936	eGFR <30 vs 60–89	3.36 (2.30–4.89)	<0.001

Logistic models adjusted for sex, age, and calendar year; eGFR 60–89 mL/min/1.73 m² was the reference. The normocytic-versus-microcytic comparison excluded 128 macrocytic cases. eGFR, estimated glomerular filtration rate; OR, odds ratio; CI, confidence interval. The added sex and age rows compare normocytic with microcytic anemia. Sex-specific models adjust for age and year; age-specific models adjust for sex, age and year. The sex and age groups overlap. Table S4 reports anemia prevalence models.

Table S3. Sensitivity analyses

Analysis	N	≥90 vs 60–89	30–59 vs 60–89	<30 vs 60–89
Primary	37,831	1.26 (1.19–1.33)	1.90 (1.75–2.07)	3.98 (3.69–4.30)
Stored counts = 1	36,188	1.26 (1.19–1.33)	1.95 (1.79–2.12)	4.12 (3.80–4.47)
Exclude eGFR >130	34,249	1.22 (1.15–1.29)	1.84 (1.69–2.00)	3.88 (3.60–4.20)
Ages 18–65	33,142	1.37 (1.28–1.46)	1.96 (1.72–2.22)	4.87 (4.36–5.43)
Flexible age adjustment	37,831	1.25 (1.18–1.32)	1.85 (1.70–2.02)	3.92 (3.62–4.24)

Entries are adjusted PRs (95% CIs) from modified Poisson models with robust standard errors, adjusted for sex, age, and calendar year. The reference was eGFR 60–89 mL/min/1.73 m². The restricted analysis retained original index records with one eligible CBC and one biochemistry record in the index month; this does not establish same-day linkage.

Table S4. Sex and age subgroup models

Subgroup	N	≥90 vs 60–89	30–59 vs 60–89	<30 vs 60–89
Men	17,670	1.10 (1.00–1.22)	1.88 (1.66–2.13)	4.73 (4.24–5.28)
Women	20,161	1.32 (1.23–1.42)	1.79 (1.60–1.99)	3.35 (3.05–3.68)
Age <50	25,115	1.51 (1.38–1.64)	1.81 (1.47–2.24)	5.03 (4.15–6.09)
Age ≥50	12,716	1.04 (0.96–1.13)	1.75 (1.60–1.92)	3.60 (3.33–3.90)
Men <50	11,641	1.59 (1.30–1.94)	2.87 (1.93–4.28)	11.99 (9.50–15.12)
Men ≥50	6,029	0.95 (0.83–1.08)	1.60 (1.41–1.83)	3.55 (3.15–4.00)
Women <50	13,474	1.47 (1.33–1.61)	1.51 (1.18–1.92)	2.31 (1.81–2.95)
Women ≥50	6,687	1.06 (0.96–1.18)	1.87 (1.64–2.12)	3.64 (3.26–4.06)

Entries are adjusted PRs (HC0 robust 95% CIs). Models adjusted for age and calendar year, and sex where applicable. The reference was eGFR 60–89 mL/min/1.73 m². The eGFR-by-sex robust Wald test allowed age and year associations to differ by sex ($P < 0.001$). Subgroup counts overlap.

Table S5. Composition of complete-case biomarker subsets

Subset	N	Anemia, n	eGFR ≥60	eGFR 30–59	eGFR <30
Kidney chemistry	5,274	1,297	5,056	162	56
Albumin–iron	6,347	1,543	6,099	189	59
HbA1c	4,546	896	4,253	218	75

All entries are counts. Subsets represent selected participants with complete data for the named biomarkers. eGFR is in mL/min/1.73 m². eGFR, estimated glomerular filtration rate; HbA1c, glycated hemoglobin.

Figure S1. Full-range association between eGFR and anemia

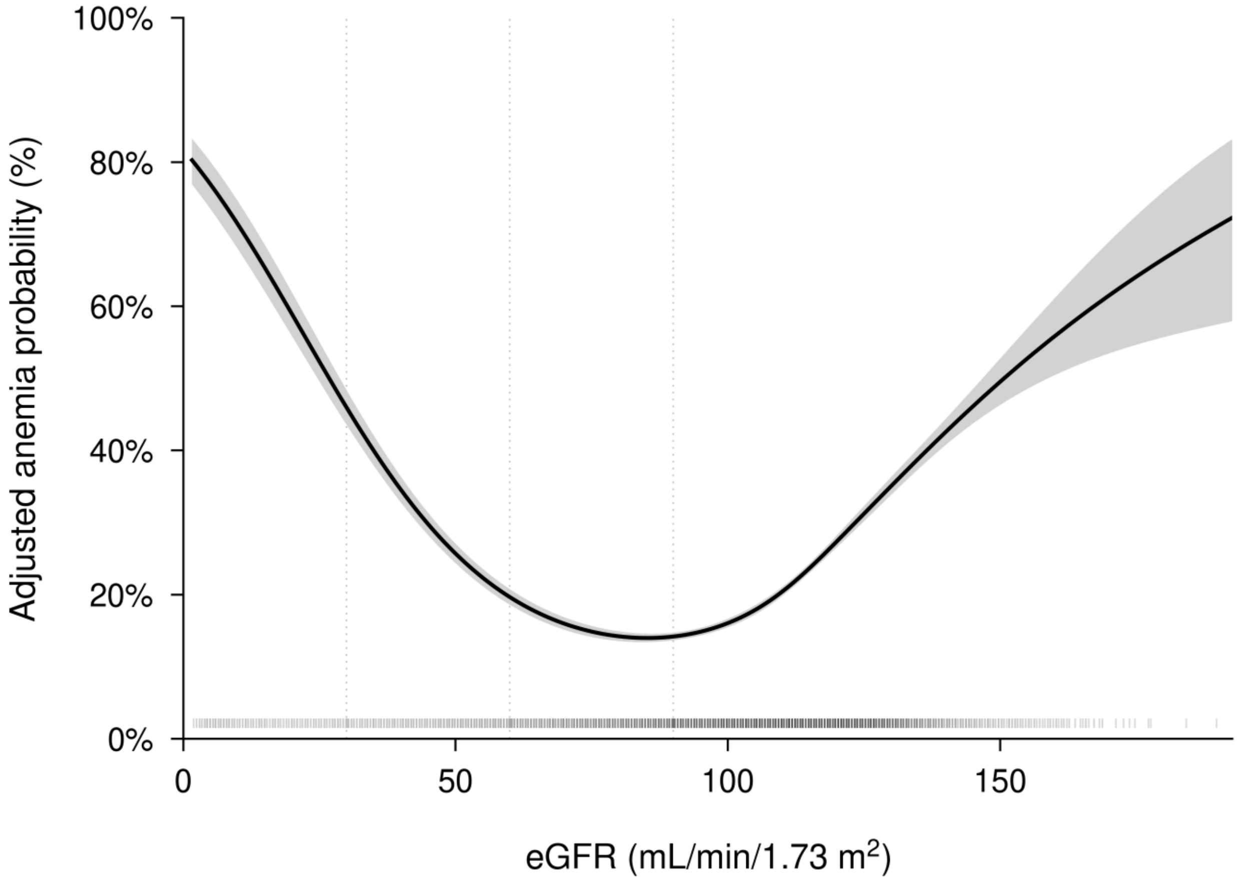

Adjusted predicted probability of anemia over the observed eGFR range (1.6–192.7 mL/min/1.73 m^2), averaged over the full cohort's covariate distribution. The black curve and grey pointwise 95% confidence band use the same model as Figure 1d. Rug marks represent counts in 0.5-unit eGFR bins, with darker marks indicating more observations. Dotted lines mark eGFR 30, 60 and 90.
